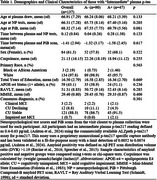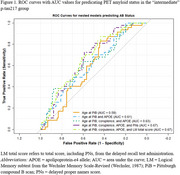# Predicting PET amyloid positivity in the intermediate plasma *p* ‐tau217 group

**DOI:** 10.1002/alz70861_108884

**Published:** 2025-12-23

**Authors:** Madeline R Hale, Kristin E Basche, Rebecca E. Langhough, Ramiro Eduardo Rea Reyes, Rachael E. Wilson, Tobey J. Betthauser, Henrik Zetterberg, Bruce P Hermann, Sterling C Johnson, Kimberly D Mueller

**Affiliations:** ^1^ Department of Communication Sciences and Disorders, University of Wisconsin‐Madison, Madison, WI USA; ^2^ Department of Medicine, University of Wisconsin‐Madison School of Medicine and Public Health, Madison, WI USA; ^3^ Wisconsin Alzheimer's Disease Research Center, University of Wisconsin‐Madison, School of Medicine and Public Health, Madison, WI USA; ^4^ Wisconsin Alzheimer’s Institute, University of Wisconsin‐Madison School of Medicine and Public Health, Madison, WI USA; ^5^ Department of Psychiatry and Neurochemistry, Institute of Neuroscience and Physiology, the Sahlgrenska Academy, University of Gothenburg, Molndal Sweden; ^6^ Clinical Neurochemistry Laboratory, Sahlgrenska University Hospital, Mölndal Sweden; ^7^ UK Dementia Research Institute, UCL Institute of Neurology, University College London, London, England UK; ^8^ Department of Neurodegenerative Disease, UCL Institute of Neurology, Queen Square, London UK; ^9^ Hong Kong Center for Neurodegenerative Diseases, Hong Kong China; ^10^ Department of Neurology, University of Wisconsin‐Madison School of Medicine and Public Health, Madison, WI USA; ^11^ Geriatric Research Education and Clinical Center (GRECC), William S. Middleton Memorial Veterans Hospital, Madison, WI USA

## Abstract

**Background:**

Plasma phosphorylated tau 217 (*p* ‐tau217) has shown promise as a less invasive, fast, and cost‐efficient method to improve early detection of Alzheimer’s disease (AD). Studies across multiple cohorts have demonstrated high sensitivity and specificity for plasma *p* ‐tau217 as a predictor of PET amyloid positivity (A+) using binary thresholds. Some have proposed a 2‐threshold approach resulting in confidently PET A‐, intermediate (risk of A+) and confidently A+. This study examines whether demographics and proper name (PN) recall, a process score from the Wechsler Logical Memory (LM) story recall test, improve predictive power relative to A+ in those with intermediate *p* ‐tau217.

**Method:**

Participants included n=137 with an intermediate plasma *p* ‐tau217 value (0.4‐0.63pg/mL) (commercial ALZpath *p* ‐tau217 assay) and the amyloid PiB‐PET scan nearest the plasma draw from the Wisconsin Registry for Alzheimer's Prevention. We used the following overlapping logistic regression models to predict PET amyloid status (A+: PiB DVR > 1.19) from the scan nearest the intermediate *p* ‐tau217 reading: age at PiB only (referred to as age); age and APOE‐e4 carrier; age, APOE‐e4 carrier, and corpulence (referred to as demographics); demographics and delayed PNs; and demographics and delayed LM total score. ROC characteristics were compared across models; differences between area under the curve (AUC) values were assessed using DeLong’s test.

**Result:**

Mean(sd) age at plasma collection was 66.91(7.29) and age at PiB scan was 68.32(7.14); 47(34.3%) were PET A+ (Table 1). PET A+ and A‐ groups did not differ significantly in NP scores or times between plasma and PET scan; 97.1% of the sample is cognitively unimpaired. AUC improved from 0.59 (age only) to 0.61 (age and APOE), 0.63 (demographics), 0.67 (demographics and delayed PNs), and 0.67 (demographics and delayed LM total score) (Figure 1). DeLong’s test indicated that these increases in AUC from the age only model or the demographics model were not significant.

**Conclusion:**

In participants with intermediate *p* ‐tau217, accuracy of predicting PET A+ status increased modestly (4%) when including existing neuropsychological data. Future work uncovering sensitive cognitive metrics may help enrich screening for PET A+ preclinical trial participants.